# 571 Effectiveness of Compression Garments with Silicone versus Compression Garments Alone on Hypertrophic Scar

**DOI:** 10.1093/jbcr/irad045.166

**Published:** 2023-05-15

**Authors:** Karen Robertson, David Wang, Khoa Tran, Ejun Yun, Katelyn Stevens, Brett Hartman

**Affiliations:** IU Health-Riley Hospital for Children, Avon, Indiana; Marian University College of Osteopathic Medicine, Indianapolis, Indiana; Indiana University School of Medicine, Indianapolis, Indiana; Indiana University School of Medicine, Indianapolis, Indiana; Indiana University School of Medicine, Indianapolis, Indiana; Indiana university/Eskenazi Health, Indianapolis, Indiana; IU Health-Riley Hospital for Children, Avon, Indiana; Marian University College of Osteopathic Medicine, Indianapolis, Indiana; Indiana University School of Medicine, Indianapolis, Indiana; Indiana University School of Medicine, Indianapolis, Indiana; Indiana University School of Medicine, Indianapolis, Indiana; Indiana university/Eskenazi Health, Indianapolis, Indiana; IU Health-Riley Hospital for Children, Avon, Indiana; Marian University College of Osteopathic Medicine, Indianapolis, Indiana; Indiana University School of Medicine, Indianapolis, Indiana; Indiana University School of Medicine, Indianapolis, Indiana; Indiana University School of Medicine, Indianapolis, Indiana; Indiana university/Eskenazi Health, Indianapolis, Indiana; IU Health-Riley Hospital for Children, Avon, Indiana; Marian University College of Osteopathic Medicine, Indianapolis, Indiana; Indiana University School of Medicine, Indianapolis, Indiana; Indiana University School of Medicine, Indianapolis, Indiana; Indiana University School of Medicine, Indianapolis, Indiana; Indiana university/Eskenazi Health, Indianapolis, Indiana; IU Health-Riley Hospital for Children, Avon, Indiana; Marian University College of Osteopathic Medicine, Indianapolis, Indiana; Indiana University School of Medicine, Indianapolis, Indiana; Indiana University School of Medicine, Indianapolis, Indiana; Indiana University School of Medicine, Indianapolis, Indiana; Indiana university/Eskenazi Health, Indianapolis, Indiana; IU Health-Riley Hospital for Children, Avon, Indiana; Marian University College of Osteopathic Medicine, Indianapolis, Indiana; Indiana University School of Medicine, Indianapolis, Indiana; Indiana University School of Medicine, Indianapolis, Indiana; Indiana University School of Medicine, Indianapolis, Indiana; Indiana university/Eskenazi Health, Indianapolis, Indiana

## Abstract

**Introduction:**

Only a few studies have looked at the effects of custom compression garments with silicone sheeting sewn into the garments versus garments alone on scar management. This retrospective study hypothesizes that garments with silicone will improve the Modified Vancouver Scar Scale (mVSS) total scores and sub-scores of pliability, vascularity and height of hypertrophic scars(HTS) when compared to garments alone.

**Methods:**

This is a retrospective study of patients that were autografted or required >21 days to heal and placed in compression garments with or without silicone between 2013 and 2020. Charts were reviewed and mVSS scores from 91 patients with 191 scar locations (134 silicone/57 non-silicone) were collected at 1,3,6,9,12 months. Descriptive statistics were used to describe the sample characteristics. The mean mVSS score and mean sub-scores for pliability, height and vascularity were computed at 1,3,6,9,12 months and reported for the silicone and non-silicone groups.

**Results:**

When comparing the two groups at 9-months (with 45% of initial scars scored), the silicone group had a greater decrease in numerical value and overall % change from 1 to 9-months as compared to the non-silicone group in all areas. The results at 12-months (with 30% of initial scars scored) demonstrated the non-silicone group had a greater decrease in numerical value and % change in height and overall score. Pliability had a 25% improvement in silicone group compared to 16% change in non-silicone group. Vascularity % change was similar with a 47% change in non-silicone group and 46% change in silicone group. The scars in silicone group that were analyzed at 12-months were consistently scored higher across prior months.

**Conclusions:**

Silicone group demonstrated improved %change in all categories at 9-months and in pliability %change at 12-months despite the decreased sample size. Pliability is improved with the use of silicone garments. Although the 12-month %change in mean for height, vascularity and total score did not show improvement over non-silicone, this reflects the return patients having significant scarring throughout treatment and needing continued interventions. These returning patients had scars in the non-silicone group as well that were also rated resulting in the disparity in the groups. The patients in the silicone group with improved scars did not continue follow-up at 12-months. Further research with focus on 9-18 month follow-up mVSS scores is warranted.

**Applicability of Research to Practice:**

Effectiveness of adding silicone to garments in scar treatment.

